# [4-(4-Meth­oxy­phen­yl)-2-(pyridin-3-yl)-1,3-thia­zol-5-yl][4-(tri­fluoro­meth­yl)phen­yl]methanone

**DOI:** 10.1107/S1600536813021259

**Published:** 2013-08-17

**Authors:** K. J. Pampa, M. M. M. Abdoh, T. R. Swaroop, K. S. Rangappa, N. K. Lokanath

**Affiliations:** a Department of Studies in Microbiology, Manasagangotri, University of Mysore, Mysore 570 006, India; b Department of Physics, Faculty of Science, An Najah National University, Nabtus, West Bank, Palestinian Territories; c Department of Studies in Chemistry, Manasagangotri, University of Mysore, Mysore 570 006, India; d Department of Studies in Physics, Manasagangotri, University of Mysore, Mysore 570 006, India

## Abstract

In the title compound, C_23_H_15_F_3_N_2_O_2_S, the thia­zole ring makes dihedral angles of 12.98 (13), 49.30 (11) and 49.83 (12)° with the pyridine ring, the meth­oxy­phenyl ring and the (tri­fluoro­meth­yl)phenyl ring, respectively. In the crystal, mol­ecules are connected *via* C—H⋯O hydrogen bonds, forming chains along [010]. There are also C—H⋯π and C—F⋯π inter­actions present, forming a three-dimensional structure.

## Related literature

For biological and other properties of thia­zoles, see: Mustafa *et al.* (2004 ▶); Sperry & Wright (2005 ▶); Zagade & Senthilkumar (2011 ▶); Narender *et al.* (2005 ▶). For the crystal structure of a related compound, see: Lu *et al.* (2006 ▶).
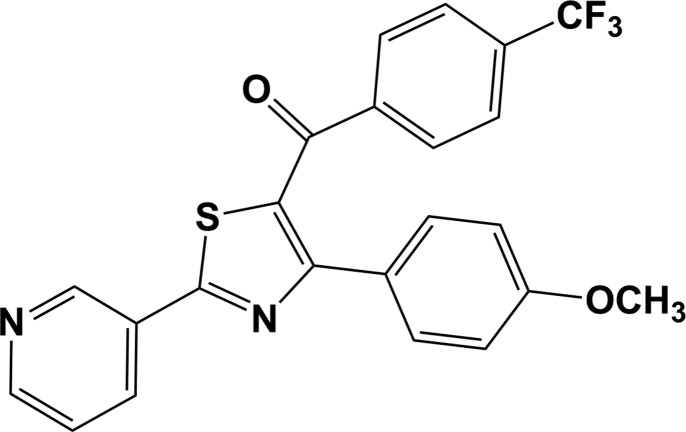


## Experimental

### 

#### Crystal data

C_23_H_15_F_3_N_2_O_2_S
*M* *_r_* = 440.44
Orthorhombic, 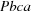

*a* = 19.4157 (5) Å
*b* = 7.6564 (2) Å
*c* = 27.4040 (7) Å
*V* = 4073.73 (18) Å^3^
*Z* = 8
Cu *K*α radiation
μ = 1.87 mm^−1^
*T* = 273 K
0.18 × 0.18 × 0.10 mm

#### Data collection

Bruker X8 Proteum diffractometer
Absorption correction: multi-scan (*SADABS*; Bruker, 2006 ▶) *T*_min_ = 0.753, *T*_max_ = 0.753
35383 measured reflections
3352 independent reflections
3140 reflections with *I* > 2σ(*I*)
*R*_int_ = 0.041

#### Refinement

*R*[*F* ^2^ > 2σ(*F* ^2^)] = 0.055
*wR*(*F* ^2^) = 0.162
*S* = 1.05
3352 reflections
281 parameters
H-atom parameters constrained
Δρ_max_ = 0.79 e Å^−3^
Δρ_min_ = −0.37 e Å^−3^

### 

Data collection: *APEX2* (Bruker, 2006 ▶); cell refinement: *SAINT* (Bruker, 2006 ▶); data reduction: *SAINT*; program(s) used to solve structure: *SHELXS97* (Sheldrick, 2008 ▶); program(s) used to refine structure: *SHELXL97* (Sheldrick, 2008 ▶); molecular graphics: *Mercury* (Macrae *et al.*, 2008 ▶); software used to prepare material for publication: *Mercury*.

## Supplementary Material

Crystal structure: contains datablock(s) global, I. DOI: 10.1107/S1600536813021259/su2625sup1.cif

Structure factors: contains datablock(s) I. DOI: 10.1107/S1600536813021259/su2625Isup2.hkl

Supplementary material file. DOI: 10.1107/S1600536813021259/su2625Isup3.cml

Additional supplementary materials:  crystallographic information; 3D view; checkCIF report

## Figures and Tables

**Table 1:** Hydrogen-bond geometry (Å, °) *Cg*1 and *Cg*2 are the centroids of the N4/C1–C3/C5/C6 and C12–C17 rings, respectively.

*D*—H⋯*A*	*D*—H	H⋯*A*	*D*⋯*A*	*D*—H⋯*A*
C13—H13⋯O19^i^	0.93	2.52	3.275 (3)	138
C5—H5⋯*Cg*1^ii^	0.93	2.93	3.597 (3)	129
C17—H17⋯*Cg*2^iii^	0.93	2.89	3.568 (2)	131
C26—F29⋯*Cg*1^iv^	1.30 (1)	3.47 (1)	4.626 (4)	149 (1)

Symmetry codes: (i) 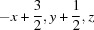; (ii) 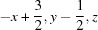; (iii) 
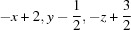
; (iv) 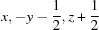.

**Fig. 1.**
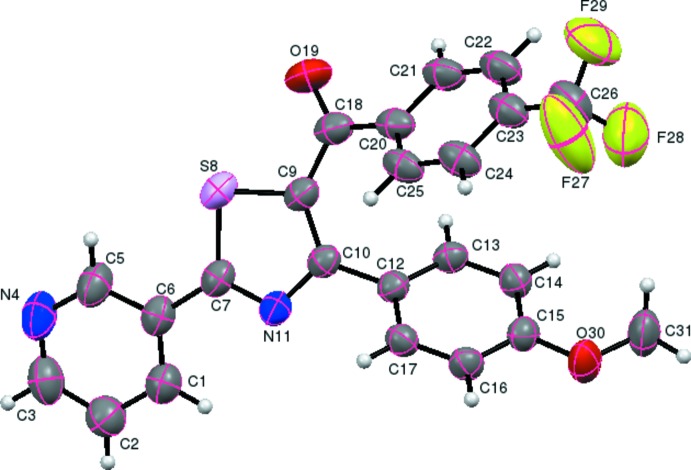
A view of the molecular structure of the title compound, with atom labelling. The displacement ellipsoids are drawn at the 50% probability level.

**Fig. 2.**
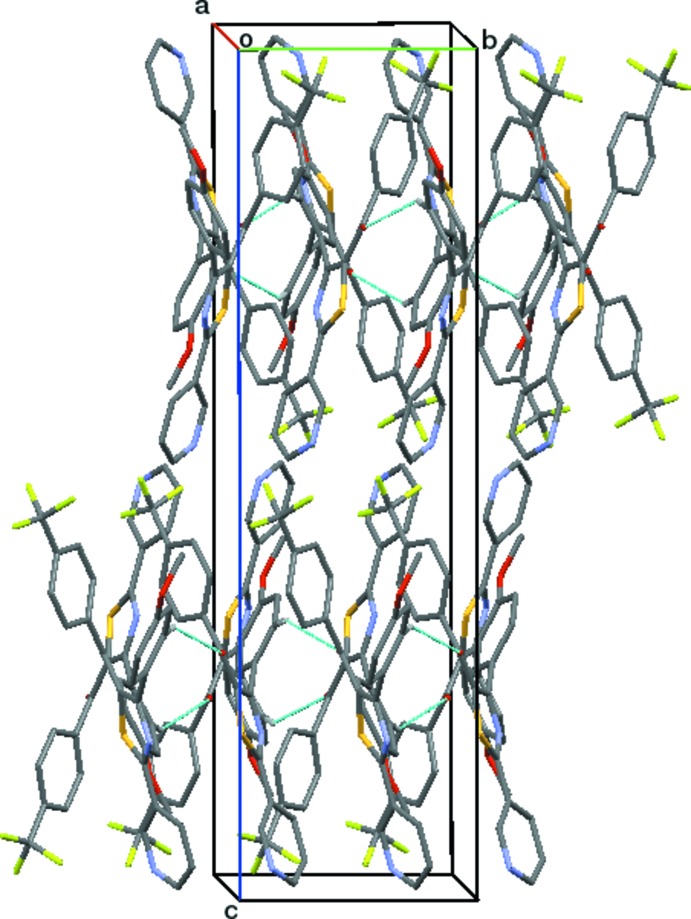
A view of the crystal packing of the title compound, viewed along the a axis. The hydrogen bonds are shown as dashed lines [see Table 1 for details; hydrogen atoms not involved in hydrogen bonding have been omitted for clarity].

## supplementary crystallographic information

### 1. Comment

Thiazoles are used in the synthesis of various drugs (Mustafa *et al.*, 2004; Sperry *et al.*, 2005), for the treatment of inflammation, hypertension, HIV infection, and as herbicides and fungicides (Zagade *et al.*, 2011; Narender *et al.*, 2005).

In the title molecule, Fig. 1, the thiazole ring makes dihedral angles of 12.98 (13)°, 49.30 (11) ° and 49.83 (12) ° with pyridine ring, the methoxy phenyl ring and the trifluoromethyl phenyl ring, respectively. The dihedral angle between the pyridine and methoxy phenyl ring is 59.60 (13) °, and with trifluoromethyl phenyl ring it is 62.78 (14) °, while the dihedral angle between the methoxy phenyl ring and trifluoromethyl phenyl ring is 33.50 (12) °. The bond lengths and bond angles of the thiazole and pyridine groups are similar to those reported for 4-Fluoro-*N*-[4-(pyridin-4-yl)thiazol-2-yl]benzamide (Lu *et al.*, 2006).

In the crystal, molecules are linked along the *a*-axis by C-H···O hydrogen bonds forming chains along [010] (Table 1 and Fig. 2). There are also C-H···π and C-F···π contacts present forming a three-dimensional structure (Table 1).

### 2. Experimental

To a solution of (*Z*)-3-(4-methoxyphenyl)-3-((pyridin-3-ylmethyl)amino)-1- (4-(trifluoromethyl)phenyl)prop-2-en-1-one (5 mmol) and DMAP (30 mmol) in dichloromethane (20 ml), thionyl chloride (30 mmol) was added at 273 K and stirred at room temperature for 3–5 h (monitored by TLC). Crushed ice was then added to quench the thionyl chloride and the reaction mixture was neutralized with 10% NaHCO_3_ solution (50 ml). The reaction was extracted with dichloromethane (2 × 50 ml) and brine (50 ml), dried over anhydrous Na_2_SO_4_ and then concentrated to give the crude title product, which was purified by column chromatography over silica gel using a hexane-EtOAc (9:1) mixture as eluent. The final product, a yellow solid, was recrystallized with ethylacetate and ethanol to form plate-like yellow crystals [HRMS calculated [M+Na] 463.0704; found 463.0700; M.p. 411-413 K]. Spectroscopic data for the title compound are available in the archived CIF.

### 3. Refinement

All the H atoms were fixed geometrically (C—H= 0.93–0.96 Å) and allowed to ride on their parent atoms with U_iso_(H) = 1.5U_eq_(C-methyl) and = 1.2U_eq_(C) for other H atoms.

### Crystal data

**Table:**

C_23_H_15_F_3_N_2_O_2_S	*F*(000) = 1808
*M**_r_* = 440.44	*D*_x_ = 1.436 Mg m^−^^3^
Orthorhombic, *P**b**c**a*	Cu *K*α radiation, λ = 1.54178 Å
Hall symbol: -P 2ac 2ab	Cell parameters from 3352 reflections
*a* = 19.4157 (5) Å	θ = 3.2–64.1°
*b* = 7.6564 (2) Å	µ = 1.87 mm^−^^1^
*c* = 27.4040 (7) Å	*T* = 273 K
*V* = 4073.73 (18) Å^3^	Plate, yellow
*Z* = 8	0.18 × 0.18 × 0.10 mm

### Data collection

**Table:**

Bruker X8 Proteum diffractometer	3352 independent reflections
Radiation source: Bruker MicroStar microfocus rotating anode	3140 reflections with *I* > 2σ(*I*)
Helios multilayer optics monochromator	*R*_int_ = 0.041
Detector resolution: 10.7 pixels mm^-1^	θ_max_ = 64.1°, θ_min_ = 3.2°
φ and ω scans	*h* = −14→22
Absorption correction: multi-scan (*SADABS*; Bruker, 2006)	*k* = −8→8
*T*_min_ = 0.753, *T*_max_ = 0.753	*l* = −26→31
35383 measured reflections	

### Refinement

**Table:**

Refinement on *F*^2^	Primary atom site location: structure-invariant direct methods
Least-squares matrix: full	Secondary atom site location: difference Fourier map
*R*[*F*^2^ > 2σ(*F*^2^)] = 0.055	Hydrogen site location: inferred from neighbouring sites
*wR*(*F*^2^) = 0.162	H-atom parameters constrained
*S* = 1.05	*w* = 1/[σ^2^(*F*_o_^2^) + (0.1015*P*)^2^ + 1.9272*P*] where *P* = (*F*_o_^2^ + 2*F*_c_^2^)/3
3352 reflections	(Δ/σ)_max_ = 0.001
281 parameters	Δρ_max_ = 0.79 e Å^−^^3^
0 restraints	Δρ_min_ = −0.37 e Å^−^^3^

### Special details

**Table:**

Experimental. Spectroscopic data for the title compound: IR (KBr, cm^-1^): 2971, 2916, 1635, 1580, 1513, 1345, 1182, 1021. ^1^H NMR (DMSO, 400 MHz) δ: 9.31 (d, J=1.9 Hz, 1H, Ar—H); 8.79 (d, J=4.6 Hz, 1.6 Hz, 1H, Ar—H), 8.49 (m, 1H, Ar—H), 7.79 (d, J=8.4 Hz, 2H, Ar—H), 7.62–7.69 (m, 3H, Ar—H), 7.45 (d,J=8.2 Hz, 2H, Ar-h), 3.70 (s, 3H, OMe).
Geometry. Bond distances, angles *etc*. have been calculated using the rounded fractional coordinates. All su's are estimated from the variances of the (full) variance-covariance matrix. The cell e.s.d.'s are taken into account in the estimation of distances, angles and torsion angles
Refinement. Refinement on *F*^2^ for ALL reflections except those flagged by the user for potential systematic errors. Weighted *R*-factors *wR* and all goodnesses of fit *S* are based on *F*^2^, conventional *R*-factors *R* are based on *F*, with *F* set to zero for negative *F*^2^. The observed criterion of *F*^2^ > σ(*F*^2^) is used only for calculating -*R*-factor-obs *etc*. and is not relevant to the choice of reflections for refinement. *R*-factors based on *F*^2^ are statistically about twice as large as those based on *F*, and *R*-factors based on ALL data will be even larger.

### Fractional atomic coordinates and isotropic or equivalent isotropic displacement parameters (Å^2^)

**Table:**

	*x*	*y*	*z*	*U*_iso_*/*U*_eq_	
S8	0.75878 (3)	0.03626 (8)	0.68759 (2)	0.0572 (2)	
F27	0.96384 (19)	−0.4401 (5)	0.93947 (8)	0.1660 (15)	
F28	0.95604 (16)	−0.1960 (4)	0.97118 (10)	0.1448 (13)	
F29	0.87856 (14)	−0.3748 (4)	0.98427 (8)	0.1311 (13)	
O19	0.70986 (9)	−0.0483 (3)	0.78419 (8)	0.0804 (8)	
O30	1.09320 (9)	0.1636 (3)	0.86314 (6)	0.0662 (6)	
N4	0.76346 (14)	0.1646 (4)	0.52908 (9)	0.0777 (9)	
N11	0.88518 (10)	0.1347 (2)	0.68325 (7)	0.0490 (6)	
C1	0.87718 (14)	0.2666 (4)	0.58435 (10)	0.0626 (8)	
C2	0.87079 (16)	0.3136 (4)	0.53576 (11)	0.0727 (10)	
C3	0.81330 (17)	0.2602 (4)	0.51042 (11)	0.0747 (10)	
C5	0.77042 (15)	0.1195 (4)	0.57561 (10)	0.0680 (9)	
C6	0.82556 (12)	0.1668 (3)	0.60518 (9)	0.0526 (7)	
C7	0.82917 (11)	0.1165 (3)	0.65670 (8)	0.0496 (7)	
C9	0.80800 (11)	0.0218 (3)	0.73973 (9)	0.0495 (7)	
C10	0.87374 (11)	0.0849 (3)	0.73068 (8)	0.0451 (6)	
C12	0.93006 (10)	0.1096 (3)	0.76629 (8)	0.0431 (6)	
C13	0.91900 (11)	0.1925 (3)	0.81070 (8)	0.0462 (7)	
C14	0.97194 (12)	0.2127 (3)	0.84395 (8)	0.0483 (7)	
C15	1.03733 (11)	0.1496 (3)	0.83313 (8)	0.0478 (7)	
C16	1.04913 (12)	0.0667 (3)	0.78865 (8)	0.0503 (7)	
C17	0.99593 (11)	0.0485 (3)	0.75569 (8)	0.0472 (7)	
C18	0.77323 (12)	−0.0467 (3)	0.78334 (10)	0.0559 (8)	
C20	0.81256 (12)	−0.1177 (3)	0.82562 (9)	0.0529 (7)	
C21	0.78670 (14)	−0.0923 (4)	0.87240 (10)	0.0620 (8)	
C22	0.82097 (15)	−0.1592 (4)	0.91228 (10)	0.0674 (9)	
C23	0.88087 (14)	−0.2555 (4)	0.90560 (9)	0.0617 (8)	
C24	0.90656 (15)	−0.2834 (3)	0.85898 (9)	0.0622 (8)	
C25	0.87225 (14)	−0.2145 (3)	0.81922 (9)	0.0563 (8)	
C26	0.9176 (2)	−0.3223 (5)	0.94866 (11)	0.0853 (11)	
C31	1.08369 (15)	0.2485 (5)	0.90855 (10)	0.0766 (10)	
H1	0.91520	0.30120	0.60250	0.0750*	
H2	0.90470	0.38000	0.52050	0.0870*	
H3	0.80940	0.29390	0.47790	0.0890*	
H5	0.73590	0.05130	0.58940	0.0820*	
H13	0.87540	0.23490	0.81820	0.0550*	
H14	0.96380	0.26860	0.87350	0.0580*	
H16	1.09270	0.02380	0.78120	0.0600*	
H17	1.00420	−0.00540	0.72590	0.0570*	
H21	0.74610	−0.02990	0.87680	0.0740*	
H22	0.80410	−0.13980	0.94360	0.0810*	
H24	0.94660	−0.34800	0.85450	0.0750*	
H25	0.88930	−0.23330	0.78790	0.0680*	
H31A	1.06850	0.36620	0.90310	0.1150*	
H31B	1.12650	0.25010	0.92610	0.1150*	
H31C	1.04970	0.18690	0.92730	0.1150*	

### Atomic displacement parameters (Å^2^)

**Table:**

	*U*^11^	*U*^22^	*U*^33^	*U*^12^	*U*^13^	*U*^23^
S8	0.0433 (4)	0.0581 (4)	0.0703 (4)	−0.0065 (2)	−0.0096 (2)	−0.0027 (3)
F27	0.220 (3)	0.201 (3)	0.0771 (14)	0.131 (3)	−0.0096 (16)	−0.0016 (15)
F28	0.156 (2)	0.170 (3)	0.1085 (18)	−0.018 (2)	−0.0384 (18)	−0.0087 (17)
F29	0.1320 (19)	0.177 (3)	0.0843 (14)	0.0050 (18)	0.0208 (13)	0.0467 (15)
O19	0.0463 (10)	0.1028 (16)	0.0922 (15)	−0.0118 (9)	0.0102 (10)	0.0018 (12)
O30	0.0535 (10)	0.0898 (13)	0.0552 (10)	0.0121 (9)	−0.0150 (8)	−0.0051 (9)
N4	0.0876 (17)	0.0781 (15)	0.0673 (14)	−0.0080 (14)	−0.0299 (13)	0.0081 (13)
N11	0.0454 (10)	0.0518 (10)	0.0498 (10)	−0.0032 (8)	−0.0068 (8)	−0.0014 (8)
C1	0.0600 (14)	0.0672 (15)	0.0605 (15)	−0.0018 (12)	−0.0087 (12)	−0.0020 (12)
C2	0.0743 (17)	0.0763 (18)	0.0675 (17)	−0.0045 (14)	−0.0033 (14)	0.0076 (14)
C3	0.091 (2)	0.0756 (18)	0.0574 (15)	0.0089 (16)	−0.0176 (15)	0.0032 (13)
C5	0.0681 (16)	0.0656 (15)	0.0702 (16)	−0.0074 (13)	−0.0243 (13)	0.0071 (13)
C6	0.0541 (13)	0.0459 (11)	0.0578 (13)	0.0037 (10)	−0.0107 (10)	−0.0037 (10)
C7	0.0468 (12)	0.0440 (11)	0.0580 (13)	−0.0008 (9)	−0.0087 (10)	−0.0031 (10)
C9	0.0418 (11)	0.0483 (12)	0.0583 (13)	−0.0028 (9)	−0.0016 (10)	−0.0047 (10)
C10	0.0429 (11)	0.0424 (10)	0.0501 (11)	−0.0009 (9)	−0.0011 (9)	−0.0039 (9)
C12	0.0410 (11)	0.0429 (10)	0.0455 (11)	−0.0031 (9)	−0.0013 (8)	0.0021 (8)
C13	0.0391 (11)	0.0488 (12)	0.0506 (12)	0.0013 (9)	0.0034 (9)	−0.0005 (9)
C14	0.0510 (12)	0.0512 (12)	0.0427 (11)	0.0021 (10)	0.0007 (9)	−0.0024 (9)
C15	0.0442 (11)	0.0535 (12)	0.0458 (11)	0.0019 (9)	−0.0060 (9)	0.0061 (9)
C16	0.0409 (11)	0.0612 (13)	0.0487 (12)	0.0071 (10)	0.0019 (9)	0.0017 (10)
C17	0.0470 (11)	0.0528 (12)	0.0418 (11)	0.0023 (9)	0.0045 (9)	−0.0006 (9)
C18	0.0459 (12)	0.0539 (13)	0.0679 (15)	−0.0066 (10)	0.0095 (11)	−0.0077 (11)
C20	0.0521 (13)	0.0472 (12)	0.0593 (13)	−0.0067 (10)	0.0148 (10)	−0.0039 (10)
C21	0.0556 (14)	0.0647 (14)	0.0657 (15)	0.0000 (12)	0.0214 (12)	−0.0077 (12)
C22	0.0723 (17)	0.0718 (16)	0.0580 (15)	−0.0044 (14)	0.0242 (13)	−0.0092 (12)
C23	0.0719 (16)	0.0597 (14)	0.0536 (14)	−0.0040 (13)	0.0165 (12)	−0.0034 (11)
C24	0.0700 (16)	0.0574 (14)	0.0592 (14)	0.0087 (12)	0.0176 (12)	−0.0002 (11)
C25	0.0673 (15)	0.0485 (13)	0.0532 (13)	0.0022 (11)	0.0205 (11)	−0.0036 (10)
C26	0.100 (2)	0.097 (2)	0.0589 (17)	0.009 (2)	0.0145 (17)	−0.0024 (16)
C31	0.0698 (17)	0.101 (2)	0.0591 (16)	0.0042 (16)	−0.0199 (13)	−0.0176 (15)

### Geometric parameters (Å, º)

**Table:**

S8—C7	1.721 (2)	C16—C17	1.379 (3)
S8—C9	1.723 (2)	C18—C20	1.490 (4)
F27—C26	1.297 (5)	C20—C21	1.390 (4)
F28—C26	1.369 (5)	C20—C25	1.387 (3)
F29—C26	1.299 (4)	C21—C22	1.378 (4)
O19—C18	1.231 (3)	C22—C23	1.389 (4)
O30—C15	1.366 (3)	C23—C24	1.388 (4)
O30—C31	1.416 (4)	C23—C26	1.471 (4)
N4—C3	1.317 (4)	C24—C25	1.382 (4)
N4—C5	1.328 (4)	C1—H1	0.9300
N11—C7	1.316 (3)	C2—H2	0.9300
N11—C10	1.373 (3)	C3—H3	0.9300
C1—C2	1.385 (4)	C5—H5	0.9300
C1—C6	1.384 (4)	C13—H13	0.9300
C2—C3	1.377 (4)	C14—H14	0.9300
C5—C6	1.391 (4)	C16—H16	0.9300
C6—C7	1.465 (3)	C17—H17	0.9300
C9—C10	1.387 (3)	C21—H21	0.9300
C9—C18	1.469 (4)	C22—H22	0.9300
C10—C12	1.478 (3)	C24—H24	0.9300
C12—C13	1.389 (3)	C25—H25	0.9300
C12—C17	1.392 (3)	C31—H31A	0.9600
C13—C14	1.382 (3)	C31—H31B	0.9600
C14—C15	1.390 (3)	C31—H31C	0.9600
C15—C16	1.393 (3)		
			
C7—S8—C9	89.45 (11)	C24—C23—C26	120.7 (3)
C15—O30—C31	117.5 (2)	C23—C24—C25	119.6 (3)
C3—N4—C5	116.3 (3)	C20—C25—C24	120.5 (2)
C7—N11—C10	111.13 (19)	F27—C26—F28	101.6 (3)
C2—C1—C6	118.4 (3)	F27—C26—F29	109.5 (3)
C1—C2—C3	118.7 (3)	F27—C26—C23	114.9 (3)
N4—C3—C2	124.4 (3)	F28—C26—F29	101.4 (3)
N4—C5—C6	124.8 (3)	F28—C26—C23	112.4 (3)
C1—C6—C5	117.5 (2)	F29—C26—C23	115.3 (3)
C1—C6—C7	120.5 (2)	C2—C1—H1	121.00
C5—C6—C7	122.0 (2)	C6—C1—H1	121.00
S8—C7—N11	114.97 (17)	C1—C2—H2	121.00
S8—C7—C6	121.99 (16)	C3—C2—H2	121.00
N11—C7—C6	123.0 (2)	N4—C3—H3	118.00
S8—C9—C10	109.87 (17)	C2—C3—H3	118.00
S8—C9—C18	116.28 (16)	N4—C5—H5	118.00
C10—C9—C18	133.8 (2)	C6—C5—H5	118.00
N11—C10—C9	114.5 (2)	C12—C13—H13	120.00
N11—C10—C12	118.04 (19)	C14—C13—H13	119.00
C9—C10—C12	127.4 (2)	C13—C14—H14	120.00
C10—C12—C13	121.51 (19)	C15—C14—H14	120.00
C10—C12—C17	119.9 (2)	C15—C16—H16	120.00
C13—C12—C17	118.57 (19)	C17—C16—H16	120.00
C12—C13—C14	120.9 (2)	C12—C17—H17	119.00
C13—C14—C15	120.0 (2)	C16—C17—H17	119.00
O30—C15—C14	124.7 (2)	C20—C21—H21	120.00
O30—C15—C16	115.6 (2)	C22—C21—H21	120.00
C14—C15—C16	119.7 (2)	C21—C22—H22	120.00
C15—C16—C17	119.7 (2)	C23—C22—H22	120.00
C12—C17—C16	121.2 (2)	C23—C24—H24	120.00
O19—C18—C9	118.6 (2)	C25—C24—H24	120.00
O19—C18—C20	119.6 (2)	C20—C25—H25	120.00
C9—C18—C20	121.8 (2)	C24—C25—H25	120.00
C18—C20—C21	118.7 (2)	O30—C31—H31A	110.00
C18—C20—C25	121.7 (2)	O30—C31—H31B	109.00
C21—C20—C25	119.6 (2)	O30—C31—H31C	110.00
C20—C21—C22	120.3 (3)	H31A—C31—H31B	109.00
C21—C22—C23	119.8 (3)	H31A—C31—H31C	109.00
C22—C23—C24	120.3 (2)	H31B—C31—H31C	109.00
C22—C23—C26	119.0 (3)		
			
C9—S8—C7—C6	−178.8 (2)	N11—C10—C12—C17	−50.4 (3)
C7—S8—C9—C10	2.23 (18)	C9—C10—C12—C13	−47.6 (4)
C7—S8—C9—C18	−179.46 (19)	C10—C12—C17—C16	−178.7 (2)
C9—S8—C7—N11	−1.13 (18)	C13—C12—C17—C16	1.1 (3)
C31—O30—C15—C16	179.4 (2)	C10—C12—C13—C14	179.1 (2)
C31—O30—C15—C14	0.0 (4)	C17—C12—C13—C14	−0.6 (3)
C5—N4—C3—C2	0.4 (5)	C12—C13—C14—C15	0.0 (3)
C3—N4—C5—C6	0.4 (5)	C13—C14—C15—C16	0.2 (3)
C10—N11—C7—S8	−0.4 (2)	C13—C14—C15—O30	179.6 (2)
C10—N11—C7—C6	177.3 (2)	O30—C15—C16—C17	−179.2 (2)
C7—N11—C10—C12	−175.5 (2)	C14—C15—C16—C17	0.2 (3)
C7—N11—C10—C9	2.2 (3)	C15—C16—C17—C12	−0.9 (3)
C2—C1—C6—C7	−178.8 (2)	O19—C18—C20—C21	−36.3 (4)
C2—C1—C6—C5	0.2 (4)	O19—C18—C20—C25	140.8 (3)
C6—C1—C2—C3	0.4 (4)	C9—C18—C20—C21	145.3 (2)
C1—C2—C3—N4	−0.8 (5)	C9—C18—C20—C25	−37.7 (3)
N4—C5—C6—C7	178.3 (3)	C18—C20—C21—C22	178.7 (3)
N4—C5—C6—C1	−0.7 (4)	C25—C20—C21—C22	1.6 (4)
C1—C6—C7—N11	−12.3 (4)	C18—C20—C25—C24	−178.0 (2)
C5—C6—C7—N11	168.7 (2)	C21—C20—C25—C24	−0.9 (4)
C5—C6—C7—S8	−13.8 (3)	C20—C21—C22—C23	−1.3 (4)
C1—C6—C7—S8	165.2 (2)	C21—C22—C23—C24	0.5 (4)
S8—C9—C18—O19	−17.7 (3)	C21—C22—C23—C26	178.6 (3)
S8—C9—C18—C20	160.72 (18)	C22—C23—C24—C25	0.1 (4)
C18—C9—C10—C12	−3.4 (4)	C26—C23—C24—C25	−177.9 (3)
S8—C9—C10—N11	−3.0 (3)	C22—C23—C26—F27	166.0 (3)
S8—C9—C10—C12	174.49 (19)	C22—C23—C26—F28	−78.4 (4)
C18—C9—C10—N11	179.1 (2)	C22—C23—C26—F29	37.1 (5)
C10—C9—C18—O19	160.1 (3)	C24—C23—C26—F27	−15.9 (5)
C10—C9—C18—C20	−21.5 (4)	C24—C23—C26—F28	99.7 (4)
N11—C10—C12—C13	129.8 (2)	C24—C23—C26—F29	−144.8 (3)
C9—C10—C12—C17	132.2 (3)	C23—C24—C25—C20	0.1 (4)

### Hydrogen-bond geometry (Å, º)

Cg1 and Cg2 are the centroids of the N4/C1–C3/C5/C6 and C12–C17 rings, respectively.

**Table:**

*D*—H···*A*	*D*—H	H···*A*	*D*···*A*	*D*—H···*A*
C13—H13···O19^i^	0.93	2.52	3.275 (3)	138
C5—H5···*Cg*1^ii^	0.93	2.93	3.597 (3)	129
C17—H17···*Cg*2^iii^	0.93	2.89	3.568 (2)	131
C26—F29···*Cg*1^iv^	1.30 (1)	3.47 (1)	4.626 (4)	149 (1)

Symmetry codes: (i) −*x*+3/2, *y*+1/2, *z*; (ii) −*x*+3/2, *y*−1/2, *z*; (iii) −*x*+2, *y*−1/2, −*z*+3/2; (iv) *x*, −*y*−1/2, *z*+1/2.
